# Radiation Therapy for Retroperitoneal Sarcomas: A Strass-Ful Situation

**DOI:** 10.3390/curroncol30010047

**Published:** 2023-01-03

**Authors:** Ahsan S. Farooqi, B. Ashleigh Guadagnolo, Devarati Mitra, Andrew J. Bishop

**Affiliations:** 1 Unit 97, Department of Radiation Oncology, The University of Texas MD Anderson Cancer Center, 1515 Holcombe Blvd., Houston, TX 77030, USA; 2 Department of Health Services Research, The University of Texas MD Anderson Cancer Center, 1515 Holcombe Blvd., Houston, TX 77030, USA

**Keywords:** retroperitoneal sarcoma, radiation therapy, surgery

## Abstract

Locoregional recurrence (LRR) is the predominant pattern of relapse and often the cause of death in patients with retroperitoneal sarcomas (RPS). As a result, reducing LRR is a critical objective for RPS patients. However, unlike soft tissue sarcomas (STS) of the superficial trunk and extremity where the benefits of radiation therapy (RT) are well-established, the role of RT in the retroperitoneum remains controversial. Historically, preoperative or postoperative RT, either alone or in combination with intraoperative radiation (IORT), was commonly justified for RPS based on extrapolation from the superficial trunk and extremity STS literature. However, long-awaited results were recently published from the European Organization for Research and Treatment of Cancer (EORTC) STRASS study of preoperative radiotherapy plus surgery versus surgery alone for patients with RPS; there was no statistical difference in the primary endpoint of abdominal recurrence-free survival. However, several subset analyses and study limitations complicate the interpretation of the results. This review explores and contextualizes the body of evidence regarding RT’s role in managing RPS.

## 1. Introduction

Soft tissue sarcomas (STS) comprise a heterogeneous group of tumors originating from mesenchymal cells, including muscle, fat, cartilage, nerve, and vascular tissue. As a result, they present at all body sites but are most commonly seen in the lower and upper extremities and less commonly in the retroperitoneum, chest wall, and head and neck regions [1]. STS comprise ~1% of all newly diagnosed malignant solid tumors, with approximately 12,000 cases seen annually in the United States [2]. Among these, retroperitoneal sarcomas (RPS) constitute approximately about 15% of all STS cases, with an average annual incidence of 2.7 per million people [3].

RPS are seen primarily in adults, with the median age reported being between 53 and 58 years, although it can also occasionally be diagnosed in children <18 years of age [4,5,6]. In adults, the most common histologic type is liposarcoma (~50–70%), which is further classified into either well-differentiated liposarcoma (WDLPS) (synonymous with atypical lipomatous tumor [ALT] when diagnosed in the extremities) and dedifferentiated liposarcoma (DDLPS) [7]. These tumors are commonly differentiated from their more benign counterpart, lipomas, when amplifications of either the *MDM2* or *CDK4* genes are present and detected by fluorescence in situ hybridization (FISH) on pathological specimens [8]. Conversely, myxoid and pleomorphic liposarcomas are less commonly seen in the retroperitoneum and tend to present in the extremities. Other common RPS histologies in adults include leiomyosarcoma (LMS), solitary fibrous tumor (SFT), malignant peripheral nerve sheath tumor (MPNST), and unclassified/undifferentiated sarcoma (formerly known as malignant fibrous histiocytoma).

WDLPS and DDLPS can often coexist and may present as a heterogenous mass within the same patient. It is also possible for a WDLPS to transform into the more-aggressive dedifferentiated phenotype, which increases the risk of distant progression. DDLPS is associated with a poorer prognosis than tumors comprised of WDLPS alone, with prior studies estimating a sixfold increase in the risk of death in the presence of a dedifferentiated component due to a 20–30% rate of developing distant metastases compared to 0% for a pure WDLPS [9,10]. Thus, accurately identifying DDLPS when diagnosing and treating RPS is critical as it informs the prognosis and may alter the treatment strategy. Positron emission tomography (PET) scans can be obtained when there is concern or possibility of a dedifferentiated component as it has been shown to have a high sensitivity and specificity for identifying DDLPS (using an SUV cut-off of 4.0) [11]. Although outside the scope of this review, it is important to be aware that neoadjuvant chemotherapy is currently being explored for patients with LMS and DDLPS RPS histologies owing to their increased risk of distant metastasis, and is the primary question being asked on the EORTC STRASS-2 (NCT04031677) prospective, randomized clinical trial [12]. 

At the time of RPS diagnosis, the patient should be referred to a sarcoma center for multidisciplinary evaluation, given the association of improved outcomes at high-volume centers. [13,14,15] Surgical resection is the mainstay in the treatment of localized RPS and is the only curative approach, with the primary oncologic goal to obtain a microscopically negative margin resection (R0). However, due to the large size of most RPS, anatomic constraints, and proximity to critical organs and vasculature, the ability to achieve an R0 resection is challenging. Most institutional series estimate gross total resection rates between 50 and 70%, and patients with R1 or R2 resections tend to have poorer survival [4,5,6,16,17]. Notably, among low/intermediate grade RPS, due to an overall low risk of distant spread, local recurrence is the leading cause of death [18]. Therefore, in extrapolating from the STS extremity literature, it was hypothesized that adding radiation therapy (RT), either in the preoperative, intraoperative, or postoperative settings, could reduce the risk of local recurrence. This review aims to summarize and contextualize the body of literature surrounding the use of RT in the management of RPS. 

## 2. Preoperative Radiation Therapy in the Management of Retroperitoneal Sarcomas

Multiple randomized trials have established that pre- or postoperative radiation therapy significantly reduces the risk of local recurrence in patients with STS of the extremities when performing a limb-sparing surgery [19,20,21]. Despite a lack of evidence for STS of the retroperitoneum, practitioners extrapolated and began to apply a similar treatment paradigm in the management of RPS, with the aim that a combined modality approach with surgery and RT may improve local relapse-free survival. One advantage of preoperative over postoperative RT is that in preoperative RT, the target (gross tumor volume [GTV]) is clearly visualized and can more accurately be defined to ensure a reproducible and precise radiation treatment plan. Additionally, lower, and thus safer, radiation doses are used in the preoperative setting. This is related to increased hypoxia in the postsurgical environment requiring higher doses to have the same efficacy [22]. Notably, the timing of RT alters the side effect profile with the potential for greater late tissue toxicity in the postoperative setting due to the higher doses used. Finally, employing RT with the tumor allows organs that may fall into the operative bed to be displaced, facilitating lower doses to nearby critical structures.

Between 1996 and 2002, a total of 94 patients with localized, primary, or recurrent, histologically confirmed RPS were enrolled in two prospective, single-arm trials open at the University of Texas MD Anderson Cancer Center (MDACC) and the University of Toronto, which sought to evaluate the tolerability and feasibility of preoperative RT [23,24] (Table 1). For patients enrolled at the University of Toronto, the planned preoperative dose was 45 Gy, which 33 of the 37 (89%) completed. The MDACC study was a dose escalation trial that aimed to determine the maximally tolerated dose by escalating from 18 Gy up to 50.4 Gy. Both studies allowed for a postoperative boost via brachytherapy or intraoperative electron therapy (IORT), and the MDACC study uniquely allowed for concurrent doxorubicin. These two studies pooled data for analysis; of the 72 patients with intermediate or high-grade RPS, the planned preoperative RT course was completed in 64 patients (93%, median dose 45 Gy), of which 54 (95%) had a macroscopically complete R0 or R1 resection [25]. Among these 54 patients, the 5-year local recurrence-free, disease-free, and overall survival rates were 60%, 46%, and 61%, respectively; the median survival was >60 months, which the authors concluded compared favorably relative to the estimated survival for RPS patients treated with surgery alone. Additionally, they reported no acute GI toxicities greater than grade 2 and no hospitalizations. However, there were grade 3 or higher hematologic toxicities in 27% of patients that received concurrent chemotherapy and postoperative complications in 39% of patients who received brachytherapy (Table 1). Therefore, the authors concluded that preoperative RT was safe and feasible and should be explored further in a prospective, randomized trial in a larger cohort of patients. 

Alongside these prospective data, other single-institutional retrospective analyses also suggested that preoperative RT may reduce the risk of local relapse [26,27]. Hull and colleagues reported that among 46 RPS patients treated with preoperative RT and a radical en bloc surgical resection, 72% were disease-free at a median follow-up of 53 months, with 10.9% demonstrating local recurrence within the RT field. Similarly, Kelly and colleagues reported outcomes of 172 patients treated with surgery alone and 32 patients treated with surgery and preoperative RT. Although there were slight imbalances in anatomic location and histologies, there were no statistical differences between groups related to tumor size, grade, and margin status. This study reported a 5-year LRFS of 91% in the RT group compared to 65% in the surgery-only group (*p* = 0.02), with no differences in disease-free survival observed. Despite these emerging data, preoperative RT in managing RPS was considered controversial and varied depending on institutional preference. 

Subsequently, a large National Cancer Database (NCDB) cohort study was reported, which used propensity score-matched analyses and included adult patients with RPS diagnosed from 2003 to 2011 to investigate local therapy practice patterns and outcomes in the US [28]. Of the 9068 patients included in their analysis, 563 patients were treated with preoperative RT, 2215 with postoperative RT, and 6290 received surgery alone. This highlighted practice patterns and demonstrated that the majority of patients with RPS in the US were being treated with surgery alone. However, when evaluating patient outcomes, both preoperative RT (HR 0.70; *p* < 0.0001) and postoperative RT (HR 0.78; *p* < 0.001) were found to be significantly associated with improved overall survival compared to surgery alone. 

As a result of these emerging data that indicated a benefit with the combined modality approach, two prospective studies were initiated to better investigate the role of RT. The ACOSOG-Z9031 (NCT00091351) trial [29] failed to accrue and was closed after enrolling less than 20 patients. However, the EORTC-62092: STRASS multicenter, randomized, phase 3 trial (NCT01344018) was successfully completed and was recently published [30] (Table 1). 

The STRASS trial randomly assigned 266 patients with non-recurrent RPS to receive either preoperative RT to a total dose of 50.4 Gy in 28 daily fractions followed by surgery or surgery alone. The primary endpoint was abdominal recurrence-free survival (ARFS), which was a composite endpoint defined as local or distant progression during receipt of RT, the patient becoming inoperable, development of peritoneal metastasis, macroscopic residual disease left in at surgery (R2), or local relapse after complete gross tumor resection. The majority of patients on the STRASS trial had either WDLPS (n = 88) or DDLPS (n = 105) histologies and less commonly LMS or other histologies (n = 72). At a median follow-up of 43.1 months, ARFS was not significantly different between cohorts (surgery plus RT: HR 1.01, 95% CI 0.71–1.44). Similarly, there was no difference in overall survival at three years (84.6% surgery alone vs. 84% with RT and surgery). Interestingly, local recurrence rates were notably higher in patients receiving surgery alone (37%) compared to those receiving preoperative RT (19.5%), and this difference was more prominently seen among patients with LPS histology. On an unplanned, exploratory analysis looking at only LPS patients, there was a 10% absolute benefit seen in reducing ARFS at three years (65% vs. 75%, HR 0.62, 95% CI 0.38–1.02). The authors concluded that STRASS was a negative trial, which does not support the routine use of preoperative RT in the management of RPS.

However, accompanying editorials and subsequent publications have raised some concerns with the interpretation of the STRASS data [31,32]. One of the common criticisms is related to the composite ARFS endpoint, particularly the inclusion of progression during radiotherapy. Statistical underpowering of the study related to histology-specific differences in outcomes were also noted. Chowdhary and Spraker editorialized concerns associated with the allowance of radiation protocol deviations from more current consensus guidelines. 

There have been two companion studies to the recent STRASS trial. The first was a combined analysis of patients enrolled in the STRASS trial and those not enrolled yet were eligible, forming the STREXIT cohort (n = 831 patients) [33]. Patients who received preoperative chemotherapy were excluded, and treatment for patients in the STREXIT cohort was determined based on consensus multidisciplinary recommendation. When combining both cohorts (n = 266 in STRASS and n = 727 in STREXIT) Callegaro and colleagues observed a statistically significant improved ARFS in patients with LPS (n = 321) who received preoperative RT (HR 0.61, 95% CI 0.42–0.89). Interestingly, the HR is similar as reported in the subset analysis in STRASS but with a tighter confidence interval.

Additionally, patients with WDLPS and G1-G2 DDLPS were found to derive the greatest benefit from RT, but those with G3 DDLPS did not, likely due to the higher rate of distant metastases in the latter subgroup. They reported no association between RT receipt and overall survival or distant-metastases-free survival. The second companion study to STRASS was conducted by Haas et al. who said that approximately 29% of RT treatment plans in the investigative arm of STRASS were non-compliant at the time of delivery, with over 66% of these plans having incorrect and non-optimal target delineation [34,35]; noncompliant RT plans were associated with a less favorable ARFS, further complicating the interpretations of the STRASS trial.

Reconciling all the data and expert opinions regarding the role of preoperative RT in RPS is difficult. However, STRASS informs us that preoperative RT should only be routinely recommended for some patients who present with de novo RPS [14,30]. Additionally, a prior study by Gronchi and colleagues reported different relapse patterns in histology and disease biology [4]. This observation was further supported by the STRASS and STREXIT data that suggest lower-grade tumors, especially LPS, may benefit most from a combined modality approach. Taking these modern prospective and past retrospective studies together, patients with de novo LPS, especially the lower grade entities, warrant a multidisciplinary discussion to determine if preoperative RT should be recommended. Other patient factors, including age, co-morbidities, and preference, should also factor into the decision-making process. Patients with de novo LMS probably should not receive RT upfront, except in special cases, given the competing risk of distant relapse. In a locally recurrent presentation, data still need to be included; however, preoperative RT should be discussed by the multidisciplinary team. Ultimately, when considering preoperative RT, the goal is to appropriately select patients whose relapse pattern is most likely local to reduce that risk. Based on the prior mentioned data, those patients that warrant discussion include LPS histology, lower grade RPS, rarer RPS histologies, recurrent RPS, and tumors at perceived increased risk for local recurrence as the predominant pattern of failure. 

## 3. Postoperative Radiation Therapy in the Management of Retroperitoneal Sarcomas

In contrast to extremity STS, the role of postoperative external beam radiation (PORT) in RPS is limited and currently not recommended [14]. The biggest limitation to using PORT is that the doses required to control microscopic disease in the postoperative setting are typically thought to be at least 60 Gy, or even higher if there is micro- or macroscopic disease left behind. Additionally, because the tumor has been resected, the volume “at risk” encompasses the bowel that has fallen into the operative bed, which increases the risk for toxicity. In one large institutional series, the 10-year complication rate for patients treated with PORT was 20% vs. 0% in patients receiving preoperative RT (*p* < 0.001), with a median dose of 60 Gy reported in the patients who had RT-related complications [36]. Similarly, adjuvant brachytherapy in RPS was also found to have higher rates of late toxicity with 4% of patients having a life-threatening toxicity [23]. Of note, this increased risk of late toxicities with the use of PORT is also observed in the extremity STS literature [37,38]. Therefore, although historically many studies reported PORT to reduce or delay time to local recurrence compared to surgery alone, including a recent meta-analysis [17,39,40], the increased late toxicities compared to preoperative RT lead many to not recommend the use of PORT in RPS outside of a clinical trial. 

## 4. Intraoperative Radiation in the Management of Retroperitoneal Sarcomas

Intraoperative radiation (IORT) is a radiation delivery technique where a single fraction of a high dose of radiation can be applied during surgery. This can either be carried out using a high dose-rate (HDR) brachytherapy afterloader, where the RT is delivered using catheters and radioactive seeds (usually Iridium-192) or through use of a miniature linear accelerator that is mounted in the operating room to allow for electron-beam RT to be directed at the tumor bed. An advantage of IORT vs. traditional external beam PORT is that the surgeon can move critical organs out of the way and attempt to expose only the tumor bed to the RT. This allows for selective dose escalation to the area at risk, thereby increasing the therapeutic ratio between the target and normal tissue. Additionally, giving a higher dose of RT in fewer fractions results in a higher “biologic” amount of RT, especially for tumors with a higher capacity for DNA repair, including most sarcomas [41,42]. For these reasons, there was enthusiasm about the use of IORT in the management of RPS when the technique was developed. The first randomized trial for RPS led by the National Cancer Institute (NCI) was conducted to evaluate IORT (20 Gy) + low dose PORT (35–40 Gy) vs. high dose PORT alone (>50 Gy) for surgically resected sarcomas of the retroperitoneum [43]. A total of 35 patients were enrolled, and the arm that received IORT was found to have significantly fewer locoregional recurrences (6 of 15) compared to the control arm of PORT alone (16 of 20). There were also fewer radiation-related abdominal complications in the IORT arm (2 of 15) compared to the control arm (10 of 20), but peripheral neuropathy rates were higher among the IORT + PORT arm (Table 2). The authors concluded that IORT should continue to be evaluated among RPS patients. Subsequent follow-up studies reported favorable local control rates within the IORT field of ~70%, but showed high rates of local failure, upwards of 60%, within the larger, yet lower dose PORT field [44,45,46]. 

Another study sought to investigate the use of IORT following preoperative RT instead. Petersen and colleagues from the Mayo Clinic reported on their experience with this strategy in 87 patients treated at their institution and observed a 59% LC rate at five years [47], but with high rates of severe GI (18%) and neuropathy (10%) toxicities. The Massachusetts General Hospital (MGH) experience also showed favorable LC (83%) in patients receiving IORT + preoperative RT compared to 61% in patients receiving preoperative RT alone [48,49]. The use of IORT remained an independent positive prognostic factor on multivariable analysis for both local control and overall survival. Based on these data, a prospective single-arm Phase I/II trial opened in Germany combining IMRT with IORT (using electrons); their preliminary analysis (n = 27) showed 72% local control at five years, yet also high rates of severe postoperative complications in 33% of their patients’ [50] (Table 2). 

## 5. Radiation Modality

There are many tools that radiation oncologists can use when treating RPS with preoperative RT, including IMRT, VMAT, and less commonly 3D-conformal radiation therapy (3D-CRT), proton therapy, or other heavy ion therapy. 3D-CRT was the first form of RT delivery that allowed for surrounding the target with a high dose of radiation (“conforming”), while simultaneously trying to minimize the dose to the surrounding normal tissues. With advances in radiation treatment delivery machines and increased computing power, a more advanced form of 3D-CRT was developed called IMRT in the late 1990s [51]. Briefly, IMRT delivers varying doses of radiation within a single beam using an inverse-planning algorithm; that allows for improved sparing of organs at risk (OAR) compared to a 3D-CRT technique. Plans using IMRT to treat RPS cases were reported to have improved conformality and reduced mean dose to the contralateral and ipsilateral kidneys, stomach, and bowel [52,53]. VMAT is similar to IMRT in that it uses an inverse planning algorithm, but the added advantage is that it allows for beam modulation as the machine rotates around the patient, not just when it is in a fixed position, as is the case with IMRT. Among RPS patients, VMAT plans, compared to IMRT, have even greater conformality, with similar dose distribution to OARs and significantly less overall treatment delivery time [54]. The recently published ASTRO guidelines on the management of STS recommend the use of IMRT/VMAT when RT is used for patients with RPS.

Compared to photon-based radiation, protons are charged particles that can also be extrinsically generated (via a cyclotron or synchrotron) and used to treat RPS patients with RT. The key advantage of protons over photons is that due to their large mass, they deposit most of their energy at the end of their range, called the Bragg Peak, beyond which there is minimal RT dose deposition [55]. They also have a denser track of ionization, so there is approximately a 10% increased biological killing efficacy compared to standard photon-based RT. For these reasons, proton therapy may have an advantage over IMRT/VMAT in select RPS cases for sparing nearby critical structures and reducing the risk of toxicity. As Chung and colleagues illustrated, intensity-modulated proton therapy (IMPT) plans have the potential to further reduce the mean dose to the liver, small bowel, and stomach when compared to standard IMRT plans if the tumor is near these OARs [56]. Limited prospective data are available to guide decisions on patient selection, and thus, potential dosimetric advantages often factor into the modality choice [57].

Like protons, carbon ions are heavy, charged particles that can be generated and used to treat patients. Carbon ion therapy also has the benefit of minimal excess RT dose deposition beyond the end of their range; therefore, it can also be used to spare OARs that are near the tumor. Since carbon ions are even heavier than protons, they are estimated to be 2 to 3 times more biologically efficacious than standard photons or even protons [58]. At this time, though, there are a very limited number of carbon ion therapy facilities in the world, with most treatments being administered in Japan and Germany [59]. Recently, Serizawa and colleagues reported on their experience in treating 24 unresectable RPS patients with carbon ions definitively; the 2-year and 5-year local control rates were 77% and 69%, respectively [60]. Additionally, they did not report any patient having > grade 2 late RT toxicities. Although these results are promising, with the limited number of patients, it is difficult to draw any meaningful conclusions. Carbon ions in RPS are currently being explored on a randomized phase II pilot trial open in Germany [61].

## 6. Radiation Considerations

**Figure 1:**
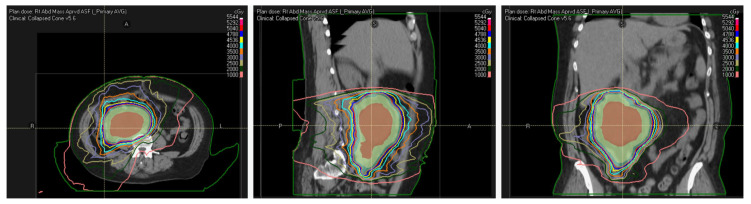
Representative axial (**A**), sagittal (**B**), and coronal (**C**) views of a VMAT radiation treatment plan generated used to preoperatively treat a patient with a right-sided retroperitoneal, recurrent, well-differentiated liposarcoma. The red color wash represents the gross tumor volume (GTV), and the yellow and blue color wash indicates the expansions used for the clinical target volume (CTV) and planning target volume (PTV), respectively. The PTV was prescribed to receive a total of 5040 cGy in 28 fractions.

Sarcoma management is complex, and it has been established that the expertise and a multidisciplinary team approach at dedicated sarcoma centers are critical to determine the optimal treatment strategy and improving clinical outcomes in patients with STS and RPS [62,63,64]. Target delineation can be challenging, as is evidenced by the recent study by Haas and colleagues illustrating that 29% of preoperative RT plans on the STRASS trial had non-compliant RT plans, with many having incorrectly delineated the target [34]. The consensus guidelines published by Baldini et al. provide a helpful criterion for target delineation, margin expansion to account for the microscopic extent of disease, and additional recommended margins for the daily set-up uncertainty [65]. As per these recommendations, when delivering preoperative RT, targets should be delineated, including the gross tumor volume (GTV) or ITV (internal target volume), clinical target volume (CTV), and planning target volumes (PTV) (Figure 1). The recommended CTV margin is an isotropic expansion of 1.5 cm around the GTV/ITV while limiting its expansion to only 5 mm within the bowel and cropping it outside of bone, muscle, or other critical OARs (kidneys, liver, etc.). It is important to note that the STRASS trial limited the CTV expansion to only 5–6 mm around the GTV, which is 1 cm less than the consensus recommendations [30]. Additional RT planning considerations are discussed in greater detail in both the RPS RT consensus and ASTRO guidelines [14,65].

A dose of 50–50.4 Gy (or GCE) at 1.8–2 Gy per fraction is recommended [14]. A strategy that is prospectively being explored is the use of a simultaneous integrated boost (SIB) using IMRT/VMAT or IMPT, which allows for a select volume to receive a higher dose of RT. Tzeng et al. reported their use of IMRT with selective dose escalation to 57.5 Gy in 25 fractions to the volume predicted to be at “highest risk” for positive surgical margins–typically, the posterior margin where the RPS abuts the muscles [66]. Among 16 patients treated with this technique, they found no severe late postoperative morbidity or mortality in any of the patients and reported an 80% actuarial LC rate at 2 years. Use of the SIB technique in the preoperative setting is currently being prospectively evaluated on a clinical trial where RPS patients receive a total dose of 63 Gy in 28 fractions with either IMRT or IMPT to the high-risk posterior retroperitoneal volume, alongside 50.4 Gy to the standard RPS treatment volumes as described above. The phase 1 arm of this trial demonstrated the dose of 63 Gy (or GCE) to be well tolerated with no significant increase in toxicity [67]. We are currently awaiting outcomes data for this trial [68].

## 7. Radiation and Immune Checkpoint Inhibition

Much research is being conducted to prospectively evaluate the role of neoadjuvant chemotherapy in the management of RPS histologies; the EORTC STRASS-2 trial aims to investigate the benefit of neoadjuvant chemotherapy in RPS that have a higher rate of DM, including LMS and DDLPS. However, other systemic agents, including immune checkpoint inhibitors (ICI), could also demonstrate activity and a favorable toxicity profile. The SARC028 phase II trial evaluated the objective response rate (ORR) of pembrolizumab against a variety of STS histologies and enrolled 86 patients with either metastatic or unresectable disease [69]. In total, 40% of UPS and 20% of LPS patients were found to have had an objective response to single-agent pembrolizumab, while no patients with LMS responded. A subsequent single institution randomized phase II trial sought to assess whether there was a difference in pathological complete response to treatment with single-agent nivolumab (a PD-1 inhibitor similar to pembrolizumab) or combination ICI therapy with both nivolumab and ipilimumab (a CTLA-4 inhibitor) for patients with either extremity undifferentiated pleomorphic sarcoma (UPS) or retroperitoneal DDLPS histologies that were undergoing preoperative RT [70]. Roland, Keung, and colleagues recently presented their preliminary findings showing a high pathological complete response (pCR) in 95% and 22.5% of patients with UPS (with the addition of preoperative RT) or DDLPS (no RT), respectively [71,72]. Historical data seem to suggest that pCR rates for STS patients treated with preoperative RT alone are low (<10%); therefore, the increased pathological response among patients receiving combination ICI + RT in this trial is an intriguing finding and warrants further evaluation on prospective clinical trials.

## 8. Conclusions

Surgical management remains the only curative modality for patients with RPS. Given all the emerging and conflicting data regarding preoperative RT for RPS, patients should be referred to a dedicated sarcoma high-volume center for multidisciplinary input. The use of preop RT should be discussed in cases with lower-grade RPS histologies, specifically G1-2 DDLPS and WDLPS, as these tumors are likely to derive the most benefit from RT. Available data do not inform the utility of RT in the recurrent setting, and thus this clinical presentation also warrants consideration and discussion. All treatment recommendations should be tailored to the individual patient, their tolerance for both acute and late toxicities, and the feasibility and potential salvage surgical options if they recur. Given the data available at this time, consensus guidelines do not recommend postoperative RT or re-irradiation. Emerging data show promise with different RT techniques and combination therapies that may help improve outcomes for this complex group of patients. Ultimately, managing RPS is complex and requires careful multidisciplinary input to optimize care for our patients.

## Figures and Tables

**Table 1:** Prospective studies evaluating the role of preoperative radiation therapy in the management of RPS.

Study	Number of Patients	Radiation Treatment Dose and Technique	Chemotherapy	Outcomes	Toxicities
MD Anderson Single-Arm Prospective Trial	35	18–50.4 Gy EBRT followed by intraoperative boost with electrons	Concurrent doxorubicin (4 mg/m^2^)	R0/R1 resections were performed in 26 of 29 (90%) evaluable patients	Grade 3–4 hematologic toxicities in 27% of patients
University of Toronto Single-Arm Prospective Trial	55	42–50 Gy EBRT followed by postoperative brachytherapy in half of the patients to doses ranging between 7.3 and 30 Gy	No	2-year OS and DFS for resected RPS patients was 88% and 80%, respectively	Postoperative Grade 3–4 toxicities in 39.1% of patients
STRASS Randomized, Prospective Phase III Trial	266	50.4 Gy in 28 fractions with EBRT	No	Median ARFS 4.5 years with RT vs. 5.0 years surgery alone (*p* = 0.95)	Serious adverse events in 24% of patients in RT group vs. 10% in surgery alone

**Table 2:** Prospective studies evaluating the role of intraoperative radiation therapy (IORT) in the management of RPS.

Study	Number of Patients	Radiation Dose and Technique	Chemotherapy	Outcomes	Toxicities
NCI Prospective, Randomized Trial	35	20 Gy IORT + 35–40 Gy PORT vs. 50–55 Gy PORT alone	Doxorubicin and Cyclophosphamide for a subset	Median OS 45 months in IORT group vs. 52 months PORT. Fewer local recurrences (6/15) in IORT arm vs. PORT-alone arm (16/20)	Peripheral neuropathy in 60% of IORT patients, vs. 5% in the PORT alone arm
German Prospective, one-armed, single center Phase I/II trial	27	Preoperative 45–50 Gy of EBRT followed by Surgery + IORT to a total dose of 10–12 Gy	No	5-year LRFS 72%, PFS 40%, and OS 74%.	33% had severe postoperative complications (9 out of 27 patients)
